# Disruptions to HIV services due to the COVID pandemic in the USA: a state-level stakeholder perspective

**DOI:** 10.1186/s12913-024-10609-9

**Published:** 2024-02-13

**Authors:** Rogério M. Pinto, Evan Hall, Vitalis Im, Carol A. Lee, Sunggeun (Ethan) Park

**Affiliations:** 1 School of Social Work, University of Michigan, 1080 South University Avenue, Ann Arbor, MI USA; 2 Department of Psychiatry, University of Michigan, Ann Arbor, MI USA

**Keywords:** HIV prevention, HIV services, COVID disruptions, Responses to COVID-19

## Abstract

**Background:**

The United States envisions a 90% reduction in HIV infections by 2030. However, the COVID-19 pandemic disrupted the HIV continuum and disproportionately affected access to social and health services for people at the highest vulnerability. This study shows how stakeholders in the State of Michigan handled disruptions and their key recommendations. As a case study, this study adds to the literature about preparedness for future pandemics.

**Methods:**

We interviewed 33 statewide Michigan HIV/AIDS Council members—practitioners, researchers, and community representatives, guiding service planning, improvement, and resource allocations, measuring group cohesiveness using a tested scale. We measured group cohesiveness as a proxy for how individual opinions reflected those of the Council as a group. We used qualitative questions to assess: (1) how the COVID-19 pandemic disrupted HIV prevention; (2) how disruptions were handled; and (3) recommendation to help address disruptions now and in the future. Using thematic analysis, we coded the interviews.

**Results:**

We found a high degree of cohesiveness. Participants agreed that the pandemic disrupted HIV prevention services (e.g., HIV testing, PrEP education, referrals to primary care, etcetera) offered by community organizations, hospital clinics, and health departments across the state. In response, they developed online and curbside services to maintain HIV services, abate social isolation, and address structural issues like lack of food and public transportation. We organized results in four categories: (1) HIV service disruptions (e.g., “*Housing for women and children who are fleeing a legal situation*”); (2) Responses to disruptions (e.g., “S*ome of them, we would say, hey, weather permitting, we’ll come out to your car*”); (3) Minoritized groups disproportionately affected (e.g., “*Especially in my community, to get people if there’s ever a vaccine, Black people are going to be the last people to take it*”); and (4) Recommendations (below).

**Conclusions:**

The pandemic unsettled and further exacerbated every aspect of HIV service provision. The main recommendation was to overhaul communication systems between government and organizations offering HIV services to mitigate disruptions and improve the chances of achieving a 90% reduction.

**Supplementary Information:**

The online version contains supplementary material available at 10.1186/s12913-024-10609-9.

## Introduction

The United States (U.S.) envisions a 90% reduction in HIV infections by the end of this decade [1]. However, the COVID-19 pandemic severely and unprecedently disrupted the lives and work of people across the globe [2, 3], including their health, stress, and life satisfaction. More specifically, the pandemic disrupted the HIV Continuum of Prevention and Care (“HIV continuum”), which connects people living with HIV (PLWH) and high-vulnerability individuals to appropriate prevention and care services, including HIV testing, pre-exposure prophylaxis (PrEP), and adherence to antiretroviral medications. A burgeoning literature started in early 2020 showing that, across the globe and particularly in the U.S., the pandemic disproportionately affected negatively those most vulnerable to HIV infections. These populations, including, Black, Indigenous, People of Color (BIPOC), men who have sex with men (MSM), people who inject/use drugs (PWID), and transgender and non-binary persons experienced a sudden disconnection from HIV continuum services [4, 5]. The initial research was based on rapid atheoretical, data collection and anecdotal accounts of how community-based organizations (CBOs), hospital clinics, and health departments in different geographic locations addressed mounting gaps in services. Currently, we have data on the services that were most disrupted and on how health service providers addressed gaps [6, 7].

We submit that the literature connecting the COVID-19 and HIV pandemic needs to expand to include local accounts of how stakeholders handled HIV services vis-à-vis COVID disruptions. The recent pandemic demonstrates how geographic location and geopolitics (e.g., shelter-in-place and shut down orders) influenced disruptions to the HIV services differently, highlighting the importance of documenting locally-based solutions and recommendations for addressing future outbreaks and disruptions. Therefore, following a community-engaged approach to research, we recruited the Michigan HIV/AIDS Council (MHAC)—a collective of practitioners, researchers, and community members across the state of Michigan, guiding the service planning, capacity improvement, and resource allocation decisions of the state for HIV resources and information. This paper explores (1) how the pandemic disrupted the HIV services with an emphasis on health inequities, (2) how HIV-serving organizations and individual providers handled the disruptions, and (3) recommendations made by the Council members.

### Impact of COVID on organizations delivering HIV continuum services and their responses

Organizations that deliver HIV services had experienced pre-pandemic matters that challenged their capacities to respond to their clients. Matters affecting organizations are both at the service provider and structural levels, such as not being able to work in the office, shutdowns, staff layoffs, institutional low morale, and fears related to COVID [8]. Community-based organizations have faced budgetary shortfalls and thus a decrease in overall employment and ability to provide essential services. Research indicates that the pandemic has disrupted both the form and function of disaster preparedness– it affected one’s ability to see people in person, to offer food and services to people required to shelter-in-place, to find volunteers to supplement service provisions, and to allocate funds while maintaining fairness and equity in the distribution of resources across populations [9].

Organizations providing HIV services implemented existing and novel approaches to HIV service disruptions, including curbside service delivery, telehealth for HIV prevention and treatment service, including PrEP prescription and maintenance [10]. However, organizations across the U.S. responded to the pandemic disruptions differently. For instance, in March 2020, most Ryan White-funded clinics in South Carolina reported partial or complete interruptions to their clinic operations hours, HIV services coverage, telehealth use, and healthcare providers’ availability. Nevertheless, these clinics reported the continuation of core HIV services, such as medication filling and testing, but stopped face-to-face counseling and social support groups. Contrastingly, Ryan White-funded clinics in Alabama suspended walk-in HIV/STI testing and community outreach events. Still, all other services (e.g., legal services, support groups, nutritional services, transportation vouchers, medical items, pet food, and personal care items) were provided through curbside services [6].

### HIV continuum disruptions and impact on key populations

The groups most affected by HIV had already been experiencing multiple personal and structural issues that the COVID pandemic further exacerbated. For example, MSM had faced challenges concerning food insecurity and housing stability before the pandemic [11]. Hepatitis C and HIV infection rates among PWID had been reduced during COVID, partly because of the increased availability of syringe service programs. However, given disruptions in services, it is still a bit too soon to know whether this reduction will be maintained now and, in the future [12]. Transgender and non-binary persons experienced job insecurity, unemployment, housing instability, and lack of health insurance prior to the pandemic [13]. There is little published research thus far on the impact of the COVID pandemic on these vulnerable populations at the intersection of HIV.

COVID-19 further aggravated these vulnerable populations’ prior conditions and ability to access HIV continuum services due to myriad changes in day-to-day domestic life, community life, or life outside of work, including childcare, caring for family members, own healthcare, food scarcity, among others [14]. As consequences, for example, research on MSM shows changes in sexual behavior, including an increase in the number of sexual partners, thus necessitating an increase in HIV prevention services during the pandemic [11]. Although there was a desired willingness among MSM to get tested for HIV and PrEP prescription/refills, access to prevention services was difficult for many [15, 16]. Similar barriers to HIV care and treatment were concurrent. MSM youth showed decreased condom usage and increased alcohol and/or substance use [17]. Therefore, there was a need for HIV services to remain open during the pandemic. Due to added demands on healthcare systems, HIV prevention programs, such as syringe exchange, were strained and even suspended [12]. Other services that became difficult to access included HIV testing, safe-use injecting equipment, and scheduling appointments with doctors and HIV counselors [18]. Adherence to antiretroviral regimens for people who inject/use drugs decreased during the pandemic alongside and a lack of optimism that PrEP was a viable prevention tool [19]. It has also been documented that, during the pandemic, transgender and non-binary individuals had less medical attention compared to pre-pandemic levels. Although there was a need to know one’s HIV status, many described substantial confusion about how to access HIV testing and other prevention services [13, 20].

### Michigan HIV/AIDS council (MHAC) as collaborative governance

By April 2020, our team had published a paper about how MHAC stakeholders forecast major HIV services disruptions at the beginning of the pandemic. Our next step was to adhere to our community-engaged research approach and collect structured interviews and data to shed light on the issues affecting the HIV services [21]. We specifically chose to work with MHAC as it represents a collaborative governance effort that “brings multiple stakeholders together in common forums with public agencies to engage in consensus-oriented decision making” [22, 23]. The goals of collaborative governance efforts, such as MHAC, are often two-fold: normative (e.g., promoting stakeholder involvement and boosting accountability) and outcome-directed (e.g., improving performance on important public policy issues) [24–26]. The Michigan Department of Health and Human Services (MIDHHS) organizes MHAC “to represent the diversity of those affected by HIV/AIDS in Michigan, maintain collaboration and coordination among prevention and care issues, and develop and sustain statewide comprehensive plans for HIV prevention and care” [23, 27]. The MHAC is a deliberative, collaborative governance vehicle to facilitate two-way communications and collaboration channels between private and public stakeholders to improve Michigan’s capacity for services and planning, and to better allocate public resources to improve the HIV continuum. The Council recruits stakeholders representing seven distinctive geographic regions from across the state with various connections to the state’s HIV continuum, including people living with HIV, representatives from AIDS service organizations (e.g., community-based organizations, non-governmental organizations), representatives from local public health agencies, and community members at large. Members serve 3-year terms and commit about 120 h per year to attend meetings and participate in committees and workgroups.

## Methods

### Sampling, recruitment, and data collection

Recruitment and data collection for this study took place approximately between August and December of 2020. The first author introduced the procedures that would be involved in the study during MHAC meetings. After that, our team sent an invitation via email to the entire membership and followed up with individual emails. Two trained research assistants conducted recruitment and the interviews using a Zoom Conferencing System. Interviews lasted between 30 and 60 min. Qualitative interviews were transcribed using Zoom transcriptions; the text for each transcribed interview was “cleaned up” for grammatical errors, missing sentences, and words before data analysis. We also asked the participants to complete the quantitative surveys (about 20 min), including basic demographic characteristics, and roles within the MHAC. In addition, the survey captured the participants’ perspectives and experience with MHAC with validated measures of group cohesion and efficacy. We interviewed 79% of the Council’s membership.

### Interview protocol

#### Semi-structured interview guide

The guide was based on the topics that had transpired in MHAC meetings when the pandemic was declared and on feedback from our early publication on potential disruptions to HIV services in Michigan and elsewhere [21]. The protocol included questions and prompts aimed to uncover (1) how the COVID-19 pandemic had disrupted the HIV services; (2) how identified disruptions had been handled; and (3) what actions participants might recommend to help address disruptions now and in the future. First, we asked participants to describe how they thought COVID had disrupted the HIV services. We prompted participants to discuss how COVID might have unearthed health inequities, particularly among people of color. Second, we asked participants to describe how they handled the identified disruptions. The third portion of the interview asked participants to briefly describe what actions they might recommend to help address disruptions now and in the future. Our prompts encouraged participants to discuss disruptions and responses related to the HIV continuum, such as HIV testing, PrEP, and primary care.

#### Group cohesiveness

We collected the survey data after the qualitative interview using paper and pencil method. We measured group cohesiveness, i.e., agreement on the purpose of MHAC’s mission, as a proxy for how individual opinions reflect those of the Council as a group. Adopted from Dobbins and Zaccaro [28], i.e., agreement on the purpose of MHAC’s mission, as a proxy for how individual opinions reflected those of the Council as a group. The cohesiveness scale stems from the leadership literature and has been adapted for myriad settings (e.g., business, health care, nursing, criminal justice, military, etc.). Group cohesiveness reflects individuals’ tendency to remain in a group or attachment to the group. Cohesiveness is influenced by group structure, leadership, and satisfaction with group. The measure includes eight statements to which participants provide their level of agreement (1 = Strongly disagree to 7 = strongly agree), such as “The members of MHAC get along well together,” “I enjoy belonging to MHAC because I am friends with many members,” and “I feel that I am really a part of the MHAC.” We calculated descriptive statistics (the mean for each item of the scale) to assess how MHAC members’ opinions and recommendations cohered as a group. We found a high degree of cohesiveness among council members (5.73; SD = 0.57). The lowest mean for any of the seven items on the scale was 5.12 (0.99) (“The members of MHAC will readily defend each other from criticism by outsiders”), and the highest was 6.36 (1.19) (“I find that I generally do not get along with other MHAC members”– reversed).

### Analytic approach and data interpretation

#### Minimizing bias and enhancing trustworthiness

Before our analysis began, we de-identified all interviews to obscure the names of participants. Each transcript was assigned a new file title. To ensure a maximum degree of “trustworthiness” [29], we held a weekly debriefing to avoid bias and fatigue. We availed ourselves of our collaboration with MHAC and held member check meetings to add validity to the findings [30]. Rigor and validation were strengthened using Dedoose, a cross-platform app for storing qualitative data, searching and retrieving text, and linking emerging themes. Because two of the authors were also members of MHAC, this process was particularly important as a deterrent to analysis bias. Their knowledge of the Council’s history, culture, and procedures enhanced data interpretation.

#### Thematic analysis

We used thematic analysis as the key method for reading, interpreting, and coding textual data. Three coders independently read and developed initial codes using open coding based on three randomly selected interviews. The coders compared their first impressions and initial codes to establish reliability. After feedback from the first author, a coding scheme began to surface. The same procedure was then repeated using another three randomly-chosen interviews and the initial codes for guidance. From this process, a coding scheme was established, which was then used to code the remaining interviews. Though the data were generated from a semi-structured interview, coders were open to finding different ideas, opinions, and observations in the text. Nonetheless, the main goal of the data analysis was to catalog disruptions due to the pandemic, responses to disruptions, and key recommendations. Therefore, we followed a coding scheme closely based on the three protocol questions described above.

#### Saturation and selecting key findings

We began to see saturation, meaning no new insights were emerging from the data until we had analyzed half of the interviews. Nonetheless, we read all interview transcripts. The results we decided to report reflect the observations and general sensibility of the entire Council. After studying all 33 interviews, an independent research assistant selected ten excerpts marked by the coders and represented the themes of the interview protocol. We used this type of selection criteria for the quotes as another step toward rigor and a way to reduce bias. As a team, we selected what we considered to be the most representative excerpts to be included in this manuscript.

## Results

### Sample description

Participants have been engaged with MHAC as voting members (sometimes for more than one term) and as volunteers (no-voting members) from less than one to more than 20 years (since its inception). Two of the authors (not interviewed for this study) were MHAC members when the pandemic was declared. The sample included 33 of 44 Michigan HIV/AIDS Council Members (response rate = 79%). The sample includes racially/ethnically diverse respondents— (52% White, 30% Black, 9% multiracial, 6% Latinx, and 3% Asian) with an average age of 42 (range: 19–62). Most participants were female (58%; male = 33%; Other = 9%) and 30% were living with HIV (*N* = 10).

### HIV services disruptions and how organizations and providers responded

Following the three areas explored in the interviews, we have provided excerpts from different participants. We used quotes that represented the ideas, opinions, and sensibilities of the majority of a council that oversees HIV service provisions in Michigan.

Participants agreed that the pandemic disrupted many services (e.g., HIV testing, PrEP education, referrals to primary care, etcetera) offered by community organizations, hospital clinics, and health departments across the state. Participants explained that the COVID-19 pandemic aggravated issues that pre-dated the pandemic and disproportionately affected minoritized groups. Participants collectively shared their inability to provide the main services that comprise the continuum of care and prevention, including but not limited to HIV testing, PrEP education and referrals to physicians who could prescribe PrEP, and day-to-day provider-client communication between providers and clients, which could help with adherence to HIV medication.

Participants highlighted how COVID affected service providers personally and hindered their ability to maintain the HIV services. One participant talked about competing responsibilities that echoed those of many others.

So, I have a two-year-old, and we were all stuck at home. My husband was also working at this time. So, we really had to maneuver around this. When my 18 [year-old] was stuck home from school… it was arguing with a grown child… about his schooling and stuff, and making sure that he was going to graduate on time, which he didn’t.

Even though service providers, and other stakeholders, had to face personal setbacks, they quickly found creative solutions for making HIV services available under difficult circumstances.

So, I actually created like a time frame for my daughter. What we did was during this time frame. So, when I would put my daughter to bed, this is when I was able to call clients…jot down my notes… everything a client needed at this time.

Participants identified several structural issues that disrupted HIV services—lack of public transportation, housing, food security, and funding insecurity around HIV services for hospitals and community-based health organizations. These issues, which pre-dated the pandemic, greatly affected providers’ ability to bill for rendered services. Similarly, uncertainty about where to send clients for HIV services affected referral-making and thus disrupted the HIV continuum. Being outside of their offices for many months, stakeholders lost connection with clients and colleagues with whom they usually collaborate (e.g., mutual referral-making) to provide HIV services.

You’re not going to come to the doctor, you have no way to get there. Those are issues which have historically been around for as long as I’ve been in this line of work…we have to fix the transportation issue; we have to fix the housing issue.

Housing for women and children who are fleeing a legal situation. So, because of COVID, these women had to be displaced, and they had to leave a safe environment and be put up into a hotel space.

The food bank service category and emergency funds category, and we had to increase those because, as we know, there were a lot of people that lost their jobs.

When this pandemic hit a lot of therapy organizations that, I was looking at, were in crisis mode of writing grants and writing letters to insurance companies as a way to stay afloat. Because a lot of insurances did not cover [pandemic-related services].

The hospital was pretty much hemorrhaging money; we weren’t able to provide any sort of care outside of code… and all those kinds of non-emergent things were put on hold, and that definitely put a little bit of a strain on the system.

### Responses to HIV services disruptions

However difficult the impacts of COVID might have been at the personal and/or professional levels, participants identified multiple responses that touched organizations, service providers, and clients alike. These responses, which may have been challenging to service providers and organizations, were developed to abate myriad issues, including inclement weather, social isolation and feelings of loneliness, replacements for in-person services, and lack of public transportation.

*We tried on different occasions to use Zoom and Google Meets to bring folks together, so they have some human contact with somebody else other than themselves.*

*Some of them, if they just can’t find a sitter for the kids, we would say, hey, weather permitting, we’ll come out to your car… we are just trying really hard to be accommodating.*

*Pretty much all clients now are either texting or calling me on my cell phone. I have to be careful because clients now call me… for example, five o’clock on a Saturday.*

*Telehealth… we were just having a conversation this morning about the issues that we’re having with that because we’re finding that throughout the entire company, only 5% of patients are actually doing the actual face or the visual communication.*

*When necessary, we arrange for folks to have an Uber for them to, you know, get to their medical appointments.*

*We’ve had to get creative with screening ahead of time on the phone. And then, of course, checking their temperature and asking them questions before [clients] come in the door.*

*We would send a consent via email. We have to actually send out a consent asking if you want to continue with our program. We would send them copies of their consents, and they would just respond back like yes or no, and then we would proceed accordingly.*

### Key recommendations to abate disruptions

Respondents’ recommendations to address future disruptions and disparities followed a common thread of responses centered around the issue of communication. For example, participants contended that health departments were not clear on which services were being offered or the format for those services being provided, whether it be virtually or in person. Most organizations continued to provide HIV testing and other services; however, communication that such services were available during the pandemic was lacking. Participants noted that communication regarding services, such as mental health, specifically directed to LGBTQ + and people of color was not clear. Hence, the main recommendation offered by participants is to overhaul all means of communication between funding agencies (e.g., Centers for Disease Control and Prevention, state and local health departments) and HIV stakeholders, especially community agencies offering HIV services across the state.

*Some [places], although they did offer HIV testing previously… now, this is complicated because on their website they provide the information that they do. But when you actually call them, they say that they actually don’t do it.*

*HIV prevention side in the state. Sometimes, communication just could have been better. I mean, especially within our health department… especially working from home and not in the building, communication could have been better about what was going on.*

*I felt like the health department was offering testing, but they didn’t…there wasn’t any messaging. Like there could have been some messaging on the radio; every slot was cheap. There could have been some billboards or could have been some type of mass communication telling people where they could go and get tested.*

*Just educating what resources and services are still available, you know, during the shutdown. And then, how to access things that clients possibly need like mental health resources because mental health is a big thing right now… just making sure that resources are reliable and inclusive, specifically for LGBTQ and people of color.*

### Minoritized groups disproportionately affected

As participants described structural issues that pre-dated the pandemic, and the disruptions to services, they also highlighted minoritized groups disproportionately affected by the pandemic. In light of the main recommendation, i.e., better communication, made by participants, it is important to emphasize these populations as they might need targeted forms of communication in case of future emergencies.

*A lot of the people that were working were not here legally. They were undocumented, and they were very nervous to talk to us. So, we had to give them that reassurance that [they could still receive services].*

*Especially in my community, to get people if there’s ever a vaccine, Black people are going to be the last people to take it.*

*It was; it still does continue to be a bit of an issue… because of living situations amongst Arabic and Hispanic populations. [COVID] is spreading quite quickly because there’s just so many people living in one residence.*

*Now, the Amish population that’s a little different. They, once they figured out that the health department was going to call them, they just completely stopped getting tested. So, they’re really only getting tested if they have an employer that’s making them.*

## Discussion

The literature suggests that COVID brought to light previous fragile structural conditions of healthcare [31], such as those identified by this study’s participants. The HIV field, from research to community action, has a rich history of adapting to political and social challenges since the beginning of the global and U.S. epidemic in the 1980s. Organizations and providers offering HIV services have been creative in addressing HIV stigma, limited funding streams, and political challenges. Nonetheless, when the World Health Organization (WHO) declared Sars-CoV-2 as the global “COVID-19 pandemic” in 2020, these same organizations and their service providers continued to help their clients again in the face of structural issues and political inaction [32].

The data from this study come from diverse HIV services stakeholders representing several regions across Michigan. MHACs format and processes engender involvement and accountability [24–26], challenged by personal and professional disruptions, many of which pre-dated the epidemic, particularly among MHAC members living with HIV. Having found a high degree of cohesiveness among study participants indicates that the qualitative data we collected reflect the opinions/sensibilities of the Council as a body that oversees HIV service provisions in Michigan. While the pandemic disrupted all HIV services (e.g., HIV testing, PrEP education, referrals to primary care, among others), the participants identified issues pre-dating the pandemic, such as poverty, housing insecurity, lack of transportation, and others. The extant literature helps to explain this study’s participants’ preoccupations with specific populations as it is known that, for the duration of the pandemic, politics enabled governments to put at risk various vulnerable groups—Black, indigenous, people of color, refugees, asylum seekers, and immigrants. Furthermore, our findings show that the effects of the COVID-19 pandemic on the disruption of HIV services were experienced differently and uniquely by each group. This indicates the need for a culturally-informed approach to recommendations like increased communication and guidelines in future emergencies.

The early months of the pandemic in 2020 were marked by shutdowns and shelter-in-place orders across the country, and then more relaxed regulations that differed across the country. By following state and local mandates, organizations offering HIV services differed in how quickly and how thoroughly they could keep their doors open and/or provide services in creative ways. Across Michigan, curbside services were widely used so that clients could safely remain in their vehicles. Like other locales, Michigan providers offered counseling, services, and support services via telephone and/or video conferencing as they developed creative ways to keep up with paperwork using email and other virtual means.

Our data show that the pandemic has been a long period of disruptions, setbacks, and triumphs. For those who did not have a car, the cold weather in Michigan posed a barrier to providing outside services during the extended winter months. Having been longer than several other states, Michigan’s stay-at-home order began on March 24, 2020, and ended on June 5, 2020. Governor Gretchen Whitmer and Health Department Director Elizabeth Hertel began to move away from initial mandates in early 2021 and encouraged people, along with service providers, to get vaccinated. Governor Whitmer vetoed legislation attempting to prevent her administration from using the public threat alert system to send out notifications regarding new mask rules. Michigan ended all restrictions on masking and gathering requirements towards the end of June 2021 [33].

This study is limited in that not all MHAC members were able to provide interviews. Although the majority of the Council participated, there might be areas of the state that were not fully represented in the sample. We also did not focus on how the pandemic affected services specifically to groups of individuals with different types and degrees of vulnerabilities– e.g., MSM, transgender people, and others. However, our research conducted through the Michigan HIV/AIDS Council is the first of its kind. Many states are required to have similar planning bodies to bring the community and lived perspectives at the state level to plan and execute the ending of HIV. Our approach to collecting local responses to the COVID-19 pandemic disruptions through interviews with MHAC members provides novel suggestions to how local responses can translate to state and national outcomes to said disruptions around HIV, especially communications. Furthermore, our data serve as a guide to historically document local responses to the COVID-19 pandemic by HIV service providers. This will catalog disruption responses in an expedited manner to propel research and innovation in light of new pandemics and world emergencies. Our study is not without impact of bias as researchers’ bias and participants’ social desirability bias are often present in qualitative research.

### Implications and recommendations

As we hoped to understand the impact of COVID-19 on HIV services, we sought information from collaborative governance comprised of multiple stakeholders [22] to develop a statewide plan for HIV prevention and care [23]. This study’s participants suggested that better communication from the Michigan Department of Health and Human Services, particularly its HIV units, might have been helpful to those providers offering HIV services. Confusion about shelter-in-place and shut down orders and HIV service discontinuity across the state were key issues identified by participants. Therefore, their recommendation is to overhaul all means of communication between funding agencies (e.g., Centers for Disease Control and Prevention, state and local health departments) and HIV stakeholders, especially community agencies offering HIV services across the state. In a personal communication with Dawn Lukomski, Section Manager, Division of HIV and STI Programs (DHSP), she explained that the Michigan Department of Health and Human Services communicated broad information to the medical providers via press releases, press conferences, and the department’s website [27]. The Division of HIV/STI Programs communicated to HIV Prevention and Care agencies via webinars, monthly check-ins with funded agencies, our website (www.michigan.gov/hivsti), newsletters, and email (Personal Communication).

## Conclusion

Our findings reflect a burgeoning literature showing that COVID-19 diminished community-based organizations’ ability to sustain the continuity of HIV services, sometimes making it uncertain that HIV infections in the U.S. will decrease by 90% within the current decade. Respondents contended that communication that clarifies specific orders and procedures that administrators and providers should follow in emergencies like the pandemic might abate the disruptions that threaten the continuity of HIV services at the dawn of a pandemic, now and in the future.

### Electronic supplementary material

Below is the link to the electronic supplementary material.

Supplementary Material 1

## Data Availability

The datasets used and/or analyzed during the current study are available from the corresponding author on reasonable request.
