# Supplementary material for: Disruptions to HIV services due to the COVID pandemic in the USA: a state-level stakeholder perspective
Source: BMC Health Serv Res. 2024 Feb 13;24:196. doi: 10.1186/s12913-024-10609-9 (PMC10865595; doi:10.1186/s12913-024-10609-9)
Supplement: Supplementary file 1 — Supplementary Material 1 [file 12913_2024_10609_MOESM1_ESM.pdf]

The Impact of COVID-19 on HIV/AIDS Services in Michigan

Qualitative Semi-Structured Interview Guide

Participants: Michigan HIV/AIDS Council (MHAC) Members

Introduction to the interview: Thank you for agreeing to participate in this research.
I am ____________ and I work at the University of Michigan, School of Social Work.

As a member MHAC, you are aware that MHAC serves as the advisory voice to HIV prevention and care for the Michigan Department of Health and Human Services (MDHHS). You also are aware that COVID-19 has disrupted the HIV Continuum of Care and Prevention across the country, and COVID-19 has also disrupted the ongoing functioning of MHAC. For example, meetings had to be rescheduled and move online.

This research aims to uncover in more specific details **(1)** how COVID-19 has disrupted the HIV Continuum of Care and Prevention; **(2)** How you have handled such disruption; and **(3)** What actions you might recommend to help address disruptions now and in the future.

Question 1a: Can you please briefly describe your connection to MHAC and what helped you decide to join?
Prompts:
A- Are you a practitioner, community member, a person living with HIV? If so, how long have lived with the virus?
B- What do you think is MHAC's main goal? What is your role in achieving this goal?
C- Are MHAC members of the same mind working toward the same goal?

Question 2a: As a [based on Question 1: practitioner, community member, researcher, etc.], can you please describe in which ways you think COVID-19 has disrupted the HIV Continuum of Care and Prevention?
Prompts:
A- Has it disrupted HIV testing? How?
B- PrEP programs? How?
C- Linking clients to primary care?
D- Helping clients stay on antiretroviral regimens?
E- Has it disrupted yours and/or your clients' ability to date, engage in social activity (including sexual activity), meet new people?
F- What else can you tell about how COVID-19 may have disrupted your life?

Question 2a: We already know that there are many racial disparities related to COVID. How do you think COVID has affected people of different races? What do you think more specifically may have cause such disparities?

Question 3a: As a [based on Question 1: practitioner, community member, researcher, etc.], can you please describe in which ways you, as a person, have handled such disruption?
Question 3b: As a, can you please describe in which ways you, as a [based on Question 1: practitioner, community member, researcher, etc.], have handled such disruption?

Prompts:
A- How have you personally and your place of work addressed disruptions in HIV testing??
B- In PrEP programs?
C- In linking clients to primary care?
D- In helping clients stay on antiretroviral regimens?
E- How have you personally addressed disruptions in social activities (including sexual activity), dating, meeting new people?

F- How have you [based on Question 1: practitioner, community member, researcher, etc.] helped others to address disruptions in their social activities (including sexual activity), dating, meeting new people?

Question 4: Can you please briefly describe what actions you might recommend to help address disruptions now and in the future. Please highlight those you have already used and which worked well, and also new strategies you think might help moving forward.

Prompts:

A- Strategies concerning HIV testing??
B- Strategies concerning PrEP programs?
C- Strategies concerning linking clients to primary care?
D- Strategies concerning helping clients stay on antiretroviral regimens?
E- Strategies concerning ways to improve social activities (including sexual activity), dating, meeting new people? Please consider personal strategies and those that might help others.
F- Any other recommendation or final thought?

Thank you very much for participating in this study. The research team will be in touch with MHAC's leadership about the analysis and interpretation of the questions. The team will make a presentation of the results in the next few months.
